# Association between Vitamin D Levels, Puberty Timing, and Age at Menarche

**DOI:** 10.3390/children10071243

**Published:** 2023-07-19

**Authors:** Valeria Calcaterra, Vittoria Carlotta Magenes, Veronica Maria Tagi, Roberta Grazi, Alice Bianchi, Hellas Cena, Gianvincenzo Zuccotti, Valentina Fabiano

**Affiliations:** 1 Department of Internal Medicine and Therapeutics, University of Pavia, 27100 Pavia, Italy; 2 Pediatric Department, Buzzi Children’s Hospital, 20154 Milano, Italy; vittoria.magenes@unimi.it (V.C.M.); veronica.tagi@unimi.it (V.M.T.); roberta.grazi@unimi.it (R.G.); alice.bianchi1@unimi.it (A.B.); gianvincenzo.zuccotti@unimi.it (G.Z.); valentina.fabiano@unimi.it (V.F.); 3 Laboratory of Dietetics and Clinical Nutrition, Department of Public Health, Experimental and Forensic Medicine, University of Pavia, 27100 Pavia, Italy; hellas.cena@unipv.it; 4 Clinical Nutrition and Dietetics Service, Unit of Internal Medicine and Endocrinology, Clinical Scientific Institutes Maugeri IRCCS, 27100 Pavia, Italy; 5 Department of Biomedical and Clinical Science, University of Milano, 20157 Milano, Italy

**Keywords:** precocious puberty, pubertal disorders, age at menarche, vitamin D, vitamin D deficiency, timing of puberty, early menarche

## Abstract

Pubertal development represents the process of physical maturation where an adolescent reaches sexual maturity and attains reproductive function. The effects of vitamin D are mainly mediated by the vitamin D receptor (VDR), which is expressed in almost all body cells, including the ovary and human pituitary gland and animal hypothalamus. Thus, vitamin D has gained great interest as pathogenic factor of pubertal disorders and fertility. This narrative review aimed to provide a broad overview of the available literature regarding the association between vitamin D levels, puberty timing, and age at menarche. A review of the data on the involvement of micronutrient deficiency, as a modifiable cause of pubertal disorders, is important for the prediction and prevention of deficiencies as well as for fertility protection and should be considered a public health priority. Reported data support that vitamin D is a regulator of neuroendocrine and ovarian physiology and, more in detail, a deficiency of vitamin D is involved in altered pubertal timing. Considering the long-term consequences of early pubertal development and early menarche, the detection of modifiable causes is crucial in preventive strategies. Future studies in humans and with an increased scale are needed to elucidate the vitamin D role in sexual maturation and puberty development.

## 1. Introduction

Vitamin D is involved in several biological functions and plays a crucial role throughout children’s growth and development [1,2]; recently, the role of vitamin D in the activation of the hypothalamic–pituitary–gonadal axis (HPG), influencing timing and progression of puberty and modulating reproductive function, has been reported [3,4].

Puberty represents the process of physical maturation where an adolescent reaches sexual maturity. There is a very wide variation in the age range of the onset of puberty, which depends on genetic, familial, ethnic, nutritional, environmental, and socioeconomic factors [5]. Although the factors underlying the individual differences in pubertal timing have not been completely identified, evidence shows that the activation of HPG depends on both genetics and environment [5].

Vitamin D deficiency prevalence is high in many parts of the world, especially among some categories of patients, such as pregnant women from ethnic minority groups in Northern Europe, Australia, and the United States, suggesting a high risk of vitamin D deficiency [6].

The effects of vitamin D are mainly mediated by the vitamin D receptor (VDR) [7], which is expressed in almost all body cells, including the ovary and human pituitary gland [8,9]. In an animal model, VDR-expressing cells were also described in the hypothalamus [10]. Thus, vitamin D has gained great interest as an influencing factor in the pathogenesis of pubertal disorders and fertility [1].

We provided a broad overview of the available literature regarding the association between vitamin D levels, puberty timing, and age at menarche. A review of the data on the involvement of micronutrient deficiency as a modifiable cause of pubertal disorders is important for the prediction and prevention of deficiencies as well as for fertility protection and should be considered a public health priority.

## 2. Methods

A narrative review on the potential connections between vitamin D deficiency and vitamin D levels and timing of puberty and age at menarche was performed. A literature search on PubMed and Embase was conducted, with language restricted to English language and published in the last 15 years. We included in our review original articles, reviews, meta-analyses, clinical practice guidelines, and commentaries regarding children aged 1–18 years. As keywords, we used precocious puberty, pubertal disorders, age at menarche, vitamin D, vitamin D deficiency, timing of puberty, and early menarche.

Starting from a total of 150 papers screened by title/abstract, the authors reviewed the full texts of relevant articles (*n* = 82). The reference list of all manuscripts was also considered to identify relevant studies.

## 3. Vitamin D: Effects, Sources, and Deficiency

**Figure 1:**
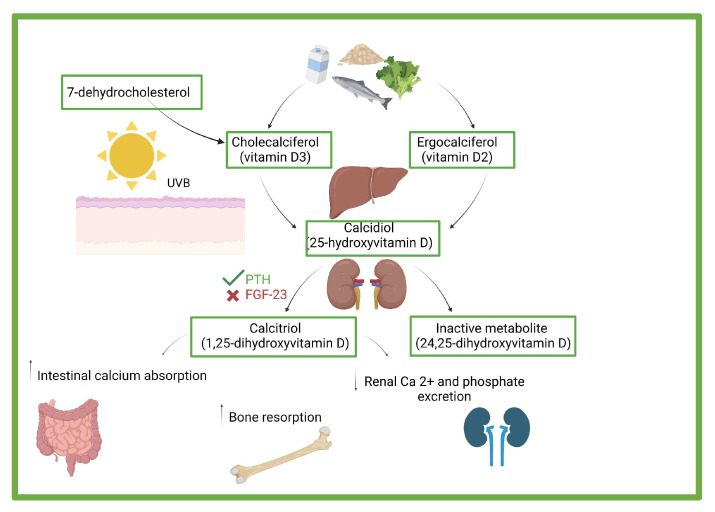
Pathways of vitamin synthesis and function. Cholecalciferol derives from animal products and supplements. It originates from the conversion of 7-dehydrocholesterol to vitamin D3 through isomerization and thermo-conversion mediated by ultraviolet B radiation in epidermal and dermal keratinocytes and fibroblasts. Ergocalciferol derives from plants and supplements. It originates from the conversion of ergosterol in plants mediated by irradiation. Calcidiol derives from 25-hydroxylation of cholecalciferol and ergocalciferol in the liver mediated by vitamin D-25-hydroxylase (CYP2R1). Calcidiol reaches the kidneys and is converted into bioactive calcitriol through hydroxylation mediated by 25(OH)D-1alfa-hydroxylase (CYP27B1), which is activated by PTH and inhibited by FGF-23. Calcitriol modulates calcium–phosphorus balance: in response to low dietary calcium intake, calcitriol induces maturation of osteoclasts and calcium–phosphorus absorption by bone and reduces renal calcium and phosphate excretion [11,12,13,14] (created with biorender.com, accessed on 10 July 2023). UVB = ultraviolet type B; PTH = parathyroid hormone; FGF = Fibroblast Growth Factors.

The term vitamin D refers to a prohormone that can be found in nature in four different forms: vitamin D3 or cholecalciferol, vitamin D2 or ergocalciferol, calcidiol (25-hydroxyVitamin D; 25OHD), and calcitriol (1,25-dihydroxyvitaminD; 1,25[OH]2D) [11]. In Figure 1, pathways of vitamin synthesis and function are shown.

Most vitamin D synthesis starts in human skin exposed to UVB. This cutaneous synthesis is influenced by sunscreen use, clothing, exposed skin area, pollution, the timing of exposure, skin pigmentation, season, latitude, and altitude. Darker skin pigmentation requires more time with sun exposure to synthesize sufficient vitamin D. UVB absorption is blocked by artificial sunscreen: a sun protection factor of 30 decreases vitamin synthesis by as much as 95%. Children require less exposure to the sun because of their body’s high surface-area-to-volume ratio. Because of a combination of these factors, children of different areas of the world such as Central and Southern Europe during fall, winter, and early spring are unable to synthesize sufficient amounts of vitamin D [2,12,15].

Only a few foods are relevant natural sources of vitamin D. The main food sources are eggs, meats, and oil-rich fishes, such as salmon and herring, which are foods rarely eaten by children. In the United States, breakfast cereal, milk, and infant formula are fortified with vitamin D. In Italy, only a few commercial yogurts and milks are fortified. Despite this tendency to supplement, the total dietary intake could be lower than the recommended intake [2,14].

Vitamin D is a crucial nutrient for maintaining a healthy mineral and bone metabolism. Indeed, vitamin D status, with calcium intake and genetic- and lifestyle-related factors, influences bone mass. It stimulates intestinal and skeletal calcium–phosphorus reabsorption and renal calcium absorption [16,17]. Moreover, vitamin D indirectly induces bone mass acquisition through development of skeletal muscle [18,19]. Recent studies, such as [20], showed a relationship between maternal vitamin D status during pregnancy, especially at 26 and 34 weeks of gestation, and fetal and neonatal bone mass.

Vitamin D deficiency as well as low calcium intake could cause nutritional rickets in children, characterized by impaired mineralization of bone and a reduction in or absence of ossification of the chondral growth plate [12]. In case of nutritional rickets, the main treatment is vitamin D administration associated with, in selected cases, calcium.

Recent research also suggests vitamin D has a beneficial effect on non-skeletal health [20,21,22]. Vitamin D regulates inflammation and modulates innate and adaptive immune responses.

Vitamin deficiency has been recently associated with the incidence and the severity of respiratory infections in children. This statement is strengthened by the evidence that children affected by rickets have an higher risk to develop pneumonia [23,24], pharyngotonsillitis, otitis media, bronchiolitis, viral wheezing, and several other types of pediatric infections, such as urinary tract infections [25], skin and soft infectious by Staphylococcus aureus [26], acute diarrhea [27], rotavirus infections [28], malaria [29], sepsis [30], tuberculosis [31], HIV [32], and hepatitis C [33].

Hypovitaminosis D could also determine asthma exacerbation and prevalence [34,35,36] as well as the development and worsening of atopic dermatitis (AD).

According to Tanpowpong et al., a vitamin D deficiency status in early life could impair immune response and mucosal integrity, thereby contributing to the pathogenesis of celiac disease. On the other hand, vitamin D deficiency in children with celiac disease and inflammatory disease could be explained by malabsorption, so it could be the consequence and not the cause of celiac disease [37].

Grant et al., Kinney et al., and Cannel et al. linked deficiency vitamin D status during pregnancy with autism [38,39,40]. Moreover, autism could determine nutritional deficiency and low dietary vitamin intake because of food selectivity associated with this condition [41].

Vitamin D deficiency could also be a cause of depression, which affects 1–6% of children worldwide. Interestingly, the vitamin D receptor is expressed in various brain areas relevant for depression pathogenesis [42,43].

Additionally, vitamin D deficiency or insufficiency has been associated with higher risks of elevated blood glucose levels, hypertension, metabolic syndrome, and obesity-related non-alcoholic fatty liver disease [44].

## 4. Assessment of Vitamin D and Risk Factors Associated to Deficiency

Considering these numerous complications associated with vitamin D deficiency, it is fundamental to detect this condition through screening strategies. According to a recent study [45], both in adults and in obese children, a questionnaire could be useful to assess vitamin D insufficiency or to monitor vitamin D status during supplementation. This questionnaire is composed of multiple-choice questions investigating factors influencing vitamin D metabolism, intake, production, or absorption. A best cut-off was established to identify more than 69% of the patients with deficiency.

Different to the adult population, in the pediatric group, no accurate threshold values have been identified to classify severity of deficiency. Further studies are needed to improve the current questionnaire and to create a dedicated simple, inexpensive, and painless tool with which to detect vitamin D deficiency in this age group [45].

As mentioned above, 25(OH)D is used to evaluate individual vitamin D status and is the best indicator of vitamin D stores. Indeed, it is the main circulating form and has a half-life of 2–3 weeks. Even if several cut-offs have been proposed, major studies classify vitamin D status as sufficiency (25(OH)D serum concentration ≥30 ng/mL), insufficiency (25(OH)D serum concentration 20–29 ng/mL), deficiency (25(OH)D serum concentration <20 ng/mL), and severe deficiency (25(OH)D serum concentration <10 ng/mL).

A meta-analysis conducted by Kumar et al. and Ginde et al. on a cohort of European studies comprising 14,971 pediatric subjects showed a hypovitaminosis D prevalence of 4–7% in 1–6-year-old subjects, 1–8% in 7–14-year-old subjects, and 12–40% in 15–18-year-old adolescents [46,47].

Numerous Italian studies showed similar data, with higher hypovitaminosis D risk in neonates and adolescents [2,12,48,49].

The most relevant risk factor for hypovitaminosis D can be distinguished in perinatal and postnatal risk factors. Among the perinatal risk factors, the most determinant are maternal vitamin D deficiency and prematurity; indeed, vitamin D is transferred from the mother to fetus across the placenta and this transfer is critical, especially during the third trimester of pregnancy [50,51,52].

Among the postnatal risk factors, the most relevant are exclusive breastfeeding because of the low vitamin D content of breast milk [53,54], low dietary intake [55,56], skin pigmentation, low sun exposure [11,57] and medications such as anticonvulsants, antiretroviral drugs, glucocorticoids, and antifungal agents [58].

According to the literature, the postnatal and pathological conditions associated with hypovitaminosis D are as follows: obesity, because of sequestration of this vitamin in fat [59,60]; a reduced concentration of vitamin D-binding protein correlated with insulin resistance [61]; an impaired hepatic 25-hydroxylation [62]; adverse dietary and lifestyle habits [63]; conditions associated with malabsorption [37]; kidney and liver diseases; and genetic disorders associated with vitamin deficiency or resistance (25-hydroxylase deficiency, 1-alpha-ydroxylase deficiency, and hereditary resistance to vitamin D) [64,65].

Because most patients with vitamin D deficiency are asymptomatic, most but not all research recommends screening the population at risk for deficiency and supplementing where appropriate [12]. Using different threshold levels of circulating total and free 25-hydroxyvitamin D for the diagnosis of vitamin D deficiency in adolescents with obesity is advised to prevent overtreatment [66].

In children with asymptomatic deficiency, supplementation with 2000 IU/day or 50,000 IU/week of D2 or D3 for 6–8 weeks is recommended. In case of the assumption of the use of drugs which interfere with vitamin D metabolism, higher doses are recommended: 2000–4000 IU/day for a minimum of 6–8 weeks [12].

Suggested vitamin D supplementation differs according to age group. Supplementation in the first year of life is essential to guarantee an adequate vitaminic status and to prevent rickets in exclusively or partially breastfed infants.

## 5. Vitamin D Levels and Precocious Puberty

The term “puberty” refers to the physical transition from sexual immaturity to sexual maturity and the consequent acquisition of the ability to reproduce. All the changes associated with puberty derive from the activation of the HPG axis. The gonadotropin-releasing hormone (GnRH) pulsatile secretion from the hypothalamus starts to increase and promotes the release of follicle-stimulating hormone (FSH) and luteinizing hormone (LH) from the anterior pituitary gland. The FSH and, in particular, the LH secretion increase in frequency and amplitude, stimulating the release of steroid hormones and gametogenesis in the gonads [5].

Puberty onset varies naturally between individuals, especially between males and females. In females, the first signs of puberty are usually seen between 8.5 and 12.5 years of age (on average 10.5 years), whereas in males these signs are seen between 9.5 and 13.5 years (on average 11.5 years) of age [67,68]. However, several factors can influence the timing of puberty onset, giving rise to precocious puberty or delayed puberty.

Genetic, endocrine, and environmental factors are involved in pubertal timing. Nutritional factors, including energy imbalance, macro and/or micronutrient food content, and dietary patterns are considered crucial factors involved in the timing of puberty [7].

As reported, the requirements of micronutrients, such as iron, zinc, calcium, and vitamin D increase during puberty. In particular, even though data are limited, a possible influence of vitamin D on the endocrine and reproductive system has been highlighted [69,70,71,72].

Several authors have suggested a possible association between vitamin D deficiency and the risk of CCP [70,71,72].

A systematic meta-analysis of six studies including 3016 precocious puberty patients and 8296 healthy individuals showed that vitamin-D-deficient subjects were more likely to develop precocious puberty [4]. Wu et al. [3] showed that lower levels of vitamin D were associated with the risk of precocious puberty (odd ratio = 2.25 and 95% CI = 1.66 and 3.04). Recently, Gan DM et al. found that a low serum 25(OH)D level is an independent risk factor for idiopathic central precocious puberty, and several characteristics of females with precocious puberty could be affected by their vitamin D status [73]. To define a cause-and-effect relationship between vitamin D levels and precocious sexual development further longitudinal studies are necessary [71].

In contrast, other studies have found no difference in vitamin D levels between patients with precocious puberty and controls [74,75]. These weak results, focused on the importance of avoiding confounding factors, such as ethnicity, overweight/obesity conditions, and the season in which the sample was collected [74]; additionally, different definitions of precocious puberty, overweight/obesity conditions, and vitamin D deficiency may also contribute to the inconstant associations.

Durá-Travé et al. showed a relationship between PTH concentrations and precocious puberty, as PTH levels in girls aged 6–8 years with precocious puberty were significantly higher in comparison with the control group, regardless of vitamin D status. These results support the hypothesis that the increase in PTH concentrations could be considered as a physiological characteristic of puberty and, in this case, with a strong correlation between PTH concentrations and bone age in the precocious puberty group [74].

In conclusion, the role of vitamin D deficiency on PP remains controversial. Further studies including more vitamin-D-sufficient subjects and avoiding confounding factors are required to determine the potential benefit of vitamin D status on the progression of puberty.

In Table 1, the main studies on the relationship between vitamin D (25OHD) levels and timing of puberty are summarized.

## 6. Vitamin D Levels and Age of Menarche

Recent evidence has suggested that vitamin D deficiency may be associated with early menarche (EM) [3,71]. However, data confirming this hypothesis are sparse in the literature since most studies are focused on vitamin-D-related food items and/or are not associated with obesity [59,60,63].

Only a few studies in the literature itself have examined the association between vitamin D status and age of menarche [71,76,77].

Villamor et al. [71] conducted a prospective study on 242 schoolgirls from Bogota, Colombia, showing that girls with low baseline 25-hydroxyVitamin D (25OHD) present more often obesity, but their early menarche persisted after adjusting for body mass index (BMI) [71]. Conversely, through an analysis of the National Health and Nutrition Examination Survey (NHANES) data from 2001 to 2010, Hua et al. observed that the apparent doubled risk of developing early menarche in girls with vitamin D deficiency vanished after adjusting for potential confounders [76].

Al-Taiar et al. [77] reported the association between 25OHD levels and age of menarche in Kuwait with a cross-sectional study and a prospective cohort study on 722 middleschool girls while also adjusting for potential confounders such as BMI, waist–height ratio, physical activity, and socioeconomic factors [77]. Even in this study, in which just the “severe deficiency category” was considered, no significant correlations between age of menarche and 25OHD status were observed [77]. The different results reported in the above-mentioned studies, performed in very different ethnic groups, might be secondary to the variability in human VDR gene polymorphisms that has been observed in different populations [78].

Data regarding the effect of vitamin D levels on EM are controversial, and further, more studies are needed to determine how vitamin D status itself influences progression of puberty [79] or how certain VDR polymorphisms affect the age of menarche.

In Table 2, the main studies that examined the association between vitamin D (25OHD) levels and age at menarche are reported.

## 7. Potential Pathogenic Mechanisms of Role of Vitamin D in Sexual Maturation

Different studies evidenced a correlation between vitamin D status, timing of menarche [71,79,80], and precocious puberty [4,72]. The pathogenesis of the relationship between vitamin D levels and sexual maturation is not yet clearly defined; however, effects on growth, obesity, VDR polymorphisms, and the HPG axis have been considered.

It is well known that anthropometric dimensions are among the determinants of puberty timing and age at menarche [75,79]. Some authors have proposed a possible effect of low vitamin D concentration on obesity development through its action on yet-unexplored adipose tissue receptors [63] and metabolizing enzymes [81]. Vitamin D is active in adipocytes at different levels and it regulates adipogenic gene expression as well as adipocyte apoptosis, interacting with membrane receptors, adaptor molecules, and nuclear coregulator proteins [81].

Zhao et al. retrospectively compared 280 girls with idiopathic CPP and 188 girls of similar ages to study serum vitamin D levels and their relationship with the risk of idiopathic CPP [72]. The authors evidenced that 91.7% of the subjects did not reach sufficient vitamin D status and the patients with CPP had significantly lower serum 25-hydroxy vitamin D (25[OH]D) levels than controls [72]. Moreover, girls affected by CPP and 25[OH]D deficiency had a higher BMI with respect to control individuals [72].

Since vitamin D is fat-soluble, and therefore sequestered within the excessive adipose tissue, given the documented earlier menarche in girls with overweight or obesity [82], an effect of vitamin D levels on age at menarche has been speculated [77]. An effect of low vitamin D concentration on EM development may also be present.

For instance, Villamor et al. examined 242 healthy girls (5–12 years old) for 30 months, showing that vitamin-D-deficient girls have an EM compared to subjects with sufficient vitamin D [71]. The authors noted that vitamin D deficiency was associated with obesity, so vitamin D status could indirectly affect the timing of menarche through its effect on obesity [70,71]. Mechanistic explanations of vitamin D deficiency and weight status affecting puberty initiation remain speculative, without a certain causal role.

A plausible role of interaction between skeletal age, menarcheal age, and vitamin D [79,83] and hormonal mediators such as estrogen and leptin levels [84] could not be excluded.

Interestingly, VDR is expressed by the HPG axis, suggesting a potential role for this receptor in the regulation of sexual maturation [4]. The polymorphism of the VDR gene, rather than the vitamin D status itself, could be responsible for early pubertal development. Calcium channel signaling may be involved in the mechanism through which VDR influences GnRH neuron function [4,84]. It is thus possible that vitamin D deficiency dysregulates GnRH neuron activity through its effects on L-type calcium channel function [4,85]. This hypothesis would be in accordance with a study conducted by Kitagawa et al., who observed that polymorphism of the VDR gene at the Apa I locus was markedly associated with early menarche [86].

Dicken et al. [80], in an animal study, showed that peripubertal vitamin D3 deficiency disrupts hypothalamic–pituitary–ovarian physiology [80]. The authors found that peripubertal vitamin D3 deficiency delayed vaginal opening without affecting the number of GnRH-immuno-positive neurons or estradiol negative feedback on gonadotropin levels during diestrus [80]. After puberty, adult females on a vitamin-D3-deficient diet had arrested follicular development and prolonged estrous cycles [80]; when vitamin-D3-deficient mice were transferred to a vitamin-D3-replete diet, the estrous cycles were restored. In this model, a hypocalcemic profile induced by vitamin D deficiency on female reproductive function could be also considered [87]; in fact, the mineral and skeletal homeostasis is crucial in normal growth and sexual maturation, and thus vitamin D and calcium homeostasis point to an important role in regulating reproductive health [88].

The effects of vitamin D3 status on the neuroendocrine axis were also evidenced by Nicholas et al., who determined whether maternal vitamin D deficiency also affects reproductive physiology in female offspring [85]. The authors found out that female mice exposed to maternal vitamin D deficiency developed irregular and prolonged estrous cycles [85]. Furthermore, females exposed to maternal vitamin D deficiency released less LH on the evening of proestrus [85]. These findings suggest that not only peripubertal vitamin D deficiency but also maternal vitamin D deficiency influences reproductive function in adult female offspring, thereby affecting hypothalamic function. In this study, a correlation between maternal vitamin D deficiency (both in utero and preweaning) and offspring puberty timing was not evidenced [85].

The direct influence of vitamin D on follicular development and ovarian steroidogenesis, thanks to the expression of vitamin D3 receptors in reproductive organs, including the ovaries, has also been investigated. Recent studies show that vitamin D3 influences anti-Müllerian hormone (AMH) concentration and thereby contributes to the modulation of ovarian reserve [89]. Studies conducted on adult women with regular menstrual cycles showed a positive correlation between the plasma concentration of 25(OH)D3 and AMH. The effect of vitamin D3 on AMH level is probably due to the presence of the VDRE sequence in the AMH gene promoter [89]. However, more studies are required to demonstrate whether the effect of vitamin D3 on AMH can play a role in pubertal timing.

**Figure 2:**
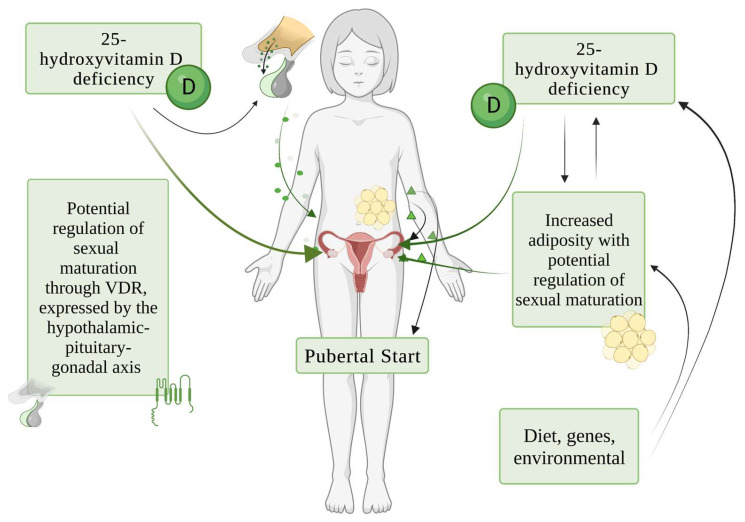
Potential mechanisms linking vitamin D and pubertal timing. VDR = vitamin D receptor.

The main potential pathogenic mechanisms of causal role of vitamin D in sexual maturation are schematically shown in Figure 2.

## 8. Long-Term Consequences of Early Pubertal Development in Females

The timing of pubertal development, especially in girls, has been deeply studied in relation to a wide range of health outcomes both physical and psychological [90].

Among physical outcomes, final height is certainly one of the most studied [90]. Indeed, during puberty there is an increment of growth velocity and finally a fusion of growth plates of long bones [5], resulting in a final stature significantly lower than the genetic target height [91,92,93]. These data conflict with the results of two retrospective studies [94,95]. Indeed, according to their works, 90% of untreated girls with early puberty achieved a normal height and both studies showed an average final height only slightly below the average of healthy peers [94,95].

Interestingly, evidence suggests that precocious puberty also seems to be associated with a higher risk of developing obesity, type 2 diabetes, hypertension, stroke, and ischemic heart disease during adulthood [90,96,97,98]. Prentice et al., in a systematic review, reported associations between earlier puberty timing (age at peak height velocity or menarche) and higher adult BMI in adult females [97]. Moreover, early menarche (<12 years) was associated with 0.34 kg/m^2^ higher BMI and doubled risk of obesity development [90].

Associations between the time of pubertal onset in girls and cardiometabolic diseases during adult life are emerging. Recently, Zhang et al. performed a meta-analysis of five and eight studies and evidenced that early (≤13 years) menarche was associated with higher insulin resistance and fasting serum insulin levels with respect to later menarche [99]. Additionally, Bubach et al. correlated early menarche (mainly ≤ 11 years) to higher blood pressure (both systolic and diastolic) and higher hypertension (>130/85 mmHg) risk among women [100]. As underlined by Chan et al. in a recent Mendelian randomization study, these associations might be causal and independent of childhood BMI [101].

Similarly, Cheng et al. and Luijken et al. reported associations between early menarche and higher risk of type 2 diabetes and/or impaired glucose tolerance [102,103] and cardiovascular diseases. It has been shown that earlier menarche was correlated with a 2–3% higher relative risk of death, per year, from all causes and ischemic heart disease [104].

Primarily because of high early exposure to estrogens, early age at menarche is also associated with a hazard to the development of any cancers, specifically estrogen-dependent cancers such as breast and endometrial cancers [98]. Moreover, in a nationally representative Brazilian Health Survey, early age at menarche (≤11 years) was correlated with a higher risk for any cancer [105], but the best-described relationship of pubertal timing with cancer was with breast cancer (BC). Indeed, Clavel-Chapelon et al. showed that the relative risk of pre- and postmenopausal BC was 7% and 3%, respectively, lower for each year of later age at menarche [106]. Moreover, the authors evidenced that BC risk in girls with age at menarche after 15 years was only 84% of that in girls with earlier menarche (before 12 years of age) [106]. Interestingly, in a recent longitudinal cohort study, Biro et al. followed up 183 girls (recruited at ages 6–7) for 14 years and reported that the correlation between early puberty and increased risk of BC might be related to increased insulin-like growth factor-1 (IGF1) levels, in addition to longer estrogen exposure.

Even though the mechanisms behind this correlation have not been clearly explained, interactions between genetic, metabolic, and hormonal factors have been suggested [107]; further studies are needed to find precise conclusions regarding this theme.

To conclude, in addition to increased risk of physical disorders later in life, earlier pubertal maturation has been associated with adverse mental health outcomes (such as depression, anxiety, eating disorders, and antisocial behaviors), higher tendency to take risky behaviors (as earlier onset of sexual activity, a higher number of sexual partners, and higher risk of substance abuse, such as alcohol and cigarettes) and lower educational achievements [108,109,110,111,112,113,114,115,116].

Pediatricians are usually the first to identify signs of early puberty; thus, they should inform and educate girls and their parents about body modifications and health and behavioral risks [117].

In view of the above-mentioned complications related to precocious puberty, it is crucial to prevent and treat precociously all modifiable causes to control long-term disease development [118].

## 9. Conclusions

The reported data support the idea that vitamin D plays a key role as a regulator of neuroendocrine and ovarian physiology and that vitamin D deficiency contributes to altered pubertal timing.

Unfortunately, evidence concerning this issue is scarce and controversial, and the majority of studies investigating the mechanisms behind this correlation are mainly on animal models. Considering the long-term consequences of early pubertal development and EM, it is crucial to detect the modifiable causes that can help in tailoring preventive strategies. Although further larger studies in humans are needed to better comprehend the role of vitamin D in sexual maturation and puberty development, there is interest in intake and supplementation at a public health level for optimal function during early life.

## Figures and Tables

**Table 1:** Summary of different studies on the relationship between Vitamin D (25OHD) levels and puberty timing.

Reference	Type of Study	Study Population	Conclusions
Lee at al. [70]	Cross-sectional study	60 girls with CPP vs. 30 controls	Significant difference in the mean serum 25OHD concentration (17.1 ± 4.5 ng/mL vs. 21.2 ± 5.0 ng/mL)
Zhao et al. [72]	Cross-sectional study	280 girls with CPP vs. 188 normal girls	The girls with CPP had significantly lower mean levels of 25OHD (19.36 ± 6.15 vs. 20.98 ± 7.60)
Liu et al. [4]	Meta-analysis	3016 PP patients vs. 8296 healthy individuals	Vitamin-D-deficient subjects were more likely to develop PP (OR = 2.02 [95% confidence interval 1.65–2.46])
Wu et al. [3]	Meta-analysis	10,755 subjects	The average serum vitamin D concentration of subjects with precocious puberty was 1.16 ng/mL, which was lower than that of the control group
Gan et al. [73]	Meta-analysis	221 girls with ICPP vs. 144 healthy girls	Serum 25(OH)D levels in the ICPP group were significantly lower than those in healthy controls (*p* < 0.001)
Duhil de Bénazé et al. [75]	Retrospective study	145 girls monitored for idiopathic CPP	The mean 25OHD concentration was 27.6 ± 17.3 ng/mL, without any correlation with puberty characteristics of the subjects
Durà-Travè et al. [74]	Cross-sectional study	78 girls with CPP vs. 137 prepubertal girls	No significant differences in 25OHD concentrations between CPP and control groups

CPP = central precocious puberty; ICPP = idiophatic central precocious puberty.

**Table 2:** Results of different studies that examined the association between vitamin D (25OHD) levels and age at menarche.

Reference	Type of Study	Study Population	Conclusions
Villamor et al. [71]	Prospective study	242 girls	Girls with low vitamin D levels had earlier menarche that persisted after adjusting for their increased body mass index
Al Taiar et al. [77]	Cross-sectional study	598 middleschool girls	No evidence for association between 25OHD level or status and age of menarche
Hua et al. [76]	Cross-sectional study	3572 females	Vitamin D status was not associated with early age of menarche after adjusting for potential confounders

## Data Availability

Not applicable.
